# Copper stress shapes the dynamic behavior of amoebae and their associated bacteria

**DOI:** 10.1093/ismejo/wrae100

**Published:** 2024-06-07

**Authors:** Yijing Shi, Lu Ma, Min Zhou, Zhili He, Yuanchen Zhao, Junyue Hong, Xinyue Zou, Lin Zhang, Longfei Shu

**Affiliations:** SCNU Environmental Research Institute, School of Environment, Guangdong Provincial Key Laboratory of Chemical Pollution and Environmental Safety & MOE Key Laboratory of Theoretical Chemistry of Environment, South China Normal University, Guangzhou 510006, China; School of Environmental Science and Engineering, Southern Marine Science and Engineering Guangdong Laboratory (Zhuhai), Guangdong Provincial Key Laboratory of Environmental Pollution Control and Remediation Technology, State Key Laboratory for Biocontrol, Sun Yat-sen University, Guangzhou 510006, China; School of Environmental Science and Engineering, Southern Marine Science and Engineering Guangdong Laboratory (Zhuhai), Guangdong Provincial Key Laboratory of Environmental Pollution Control and Remediation Technology, State Key Laboratory for Biocontrol, Sun Yat-sen University, Guangzhou 510006, China; School of Environmental Science and Engineering, Southern Marine Science and Engineering Guangdong Laboratory (Zhuhai), Guangdong Provincial Key Laboratory of Environmental Pollution Control and Remediation Technology, State Key Laboratory for Biocontrol, Sun Yat-sen University, Guangzhou 510006, China; School of Environmental Science and Engineering, Southern Marine Science and Engineering Guangdong Laboratory (Zhuhai), Guangdong Provincial Key Laboratory of Environmental Pollution Control and Remediation Technology, State Key Laboratory for Biocontrol, Sun Yat-sen University, Guangzhou 510006, China; School of Environmental Science and Engineering, Southern Marine Science and Engineering Guangdong Laboratory (Zhuhai), Guangdong Provincial Key Laboratory of Environmental Pollution Control and Remediation Technology, State Key Laboratory for Biocontrol, Sun Yat-sen University, Guangzhou 510006, China; School of Environmental Science and Engineering, Southern Marine Science and Engineering Guangdong Laboratory (Zhuhai), Guangdong Provincial Key Laboratory of Environmental Pollution Control and Remediation Technology, State Key Laboratory for Biocontrol, Sun Yat-sen University, Guangzhou 510006, China; School of Environmental Science and Engineering, Southern Marine Science and Engineering Guangdong Laboratory (Zhuhai), Guangdong Provincial Key Laboratory of Environmental Pollution Control and Remediation Technology, State Key Laboratory for Biocontrol, Sun Yat-sen University, Guangzhou 510006, China; School of Environmental Science and Engineering, Southern Marine Science and Engineering Guangdong Laboratory (Zhuhai), Guangdong Provincial Key Laboratory of Environmental Pollution Control and Remediation Technology, State Key Laboratory for Biocontrol, Sun Yat-sen University, Guangzhou 510006, China

**Keywords:** copper stress, soil amoebae, *Paraburkholderia* colonization, symbiosis, cell fate switching, pathogen transmission

## Abstract

Amoeba-bacteria interactions are prevalent in both natural ecosystems and engineered environments. Amoebae, as essential consumers, hold significant ecological importance within ecosystems. Besides, they can establish stable symbiotic associations with bacteria. Copper plays a critical role in amoeba predation by either killing or restricting the growth of ingested bacteria in phagosomes. However, certain symbiotic bacteria have evolved mechanisms to persist within the phagosomal vacuole, evading antimicrobial defenses. Despite these insights, the impact of copper on the symbiotic relationships between amoebae and bacteria remains poorly understood. In this study, we investigated the effects of copper stress on amoebae and their symbiotic relationships with bacteria. Our findings revealed that elevated copper concentration adversely affected amoeba growth and altered cellular fate. Symbiont type significantly influenced the responses of the symbiotic relationships to copper stress. Beneficial symbionts maintained stability under copper stress, but parasitic symbionts exhibited enhanced colonization of amoebae. Furthermore, copper stress favored the transition of symbiotic relationships between amoebae and beneficial symbionts toward the host’s benefit. Conversely, the pathogenic effects of parasitic symbionts on hosts were exacerbated under copper stress. This study sheds light on the intricate response mechanisms of soil amoebae and amoeba-bacteria symbiotic systems to copper stress, providing new insights into symbiotic dynamics under abiotic factors. Additionally, the results underscore the potential risks of copper accumulation in the environment for pathogen transmission and biosafety.

## Introduction

Symbiotic relationships pervade natural ecosystems and occupy a central role in ecological functioning and evolutionary dynamics. Hosts and their symbionts exchange goods and services through intimate physical or chemical contact [1] and even acquire modifications in genomes and structure [2]. A classic example is that the mitochondria of eukaryotes were considered to originate from the endosymbiosis of bacteria [3, 4]. These symbiotic interactions can be beneficial, neutral, or harmful to the host organism. However, the interactions between hosts and symbionts are not simple binary systems. Instead, they can shift along the parasitic–mutualist continuum in response to environmental changes and ecological context during evolutionary trajectories [5, 6]. Therefore, investigating the successional dynamics of symbiotic relationships will provide new insights into the assembly of biological communities and the emergence of new functions.

Symbiotic interactions with bacteria can be widely found in eukaryotic hosts including animals, plants, and protists [7–9]. Amoebae, a major clade of protists, exist in a variety of ecosystems where they are known as bacterial predators and provide significant ecosystem services as consumers [10]. However, various bacteria have developed strategies to survive amoeba phagocytosis [11, 12], and reside intracellularly to survive harsh conditions [13, 14]. Moreover, certain bacterial pathogens exhibit intracellular multiplication within amoebae, acquiring pathogenicity and virulence through this intra-amoeba growth [15, 16]. Overall, amoebae and bacteria are involved in highly complex interactions, which were regulated by the virulence properties of both amoebae and bacteria, as well as the prevailing environmental conditions [17]. This system provides a valuable model to study the dynamics of symbiosis.

*Dictyostelium discoideum* is a soil-dwelling amoeba with a unique life cycle encompassing both unicellular and multicellular stages [18]. During the unicellular phase, *D*. *discoideum* cells prey on bacteria by phagocytosis and proliferate by binary fission. Upon depletion of the available food supply, *D*. *discoideum* cells aggregate and develop into a mature fruiting body [18]. Wild *D. discoideum* strains commonly host one or more culturable endosymbiotic bacteria, which can serve as food, symbionts, or pathogens. Among these, species within the genus *Paraburkholderia* are frequently encountered [19, 20]. These *Paraburkholderia* strains exert context-dependent effects on their amoeba hosts, ranging from mutualistic to pathogenic interactions [21]. Specifically, *Paraburkholderia* can initiate a farming symbiosis of amoeba hosts and food bacteria to increase spore productivity under food-scarce conditions [20, 22], but also have detrimental effects on the hosts in food-rich conditions [23]. In addition, the *D*. *discoideum-Paraburkholderia* interaction does not occur randomly through phagocytosis, evidence showed that *Paraburkholderia* symbionts can use chemotaxis to find their hosts actively and form stable symbiotic relationships [24, 25].

Copper is an important element with broad-spectrum antibacterial properties that has been utilized in various fields [26, 27]. This antibacterial mechanism has also been uncovered in protists and vertebrates, which were used to kill the ingested bacteria in phagosomes [28]. In the predatory relationship of amoebae and bacteria, copper plays an important role in protistan grazing and this predation mechanism creates selective pressure on bacteria to promote the bacterial copper resistance [29, 30]. However, some bacteria such as *Paraburkholderia* persist within the phagosomal vacuole by subverting the antimicrobial mechanisms [31]. Despite these insights, further exploration is needed to understand the impact of copper on the symbiotic relationships between amoebae and bacteria [32]. *Paraburkholderia agricolaris* and *Paraburkholderia hayleyella* are two main species associated with *D*. *discoideum* which show different infection patterns to hosts [21, 33], with *P. hayleyella* being most detrimental and *P. agricolaris* being more modest in general [23, 34]. Comparative genomics analysis has revealed that *P. hayleyella* harbors a smaller genome size and lower GC content compared with *P. agricolaris*, a common pattern associated with long-term symbiotic interactions [35]. Given their distinct infection phenotypes and symbiotic histories, *P. hayleyella* and *P. agricolaris* are classified as parasitic and beneficial symbionts, respectively. However, only one-third of wild *D*. *discoideum* strains are detected with culturable endosymbiotic bacteria [20, 22], indicating that even though *Paraburkholderia* could successfully colonize other non-native hosts; the association history differed between primitive symbiosis and non-primitive symbiosis. In primitive symbiosis, long-term interaction has led the native amoeba hosts to evolve mechanisms to tolerate their symbionts [23]. Moreover, the genomes of *Paraburkholderia* symbionts have increased secretion systems and motility genes that may mediate interactions with their hosts [35]. These adaptive evolutions show that *Paraburkholderia* symbionts have a long history of co-evolution with their native hosts. Differences in symbiont types and evolution histories may influence the response of symbiotic systems to environmental stresses.

Considering the inhibitory effect of excessive copper on amoebae [36] and the anti-predation mechanisms employed by *Paraburkholderia*, we hypothesized that excessive copper would enhance the formation of the symbiotic relationships between amoebae and bacteria. To explore the effects of symbiont type and coevolution history on the responses of symbiotic relationships, we constructed six symbiotic systems using two symbionts with three amoeba hosts (native and non-primitive hosts). Exposure experiments, chemotaxis experiments, and transcriptome sequencing were conducted to address the following questions: (i) How does copper stress affect amoeba’s fitness? (ii) How does copper stress alter the symbiotic relationships between amoebae and *Paraburkholderia* symbionts? (iii) How do symbiont types and evolutionary time influence the response of symbiotic relationships to copper stress?

## Materials and methods

### Cultivation of amoebae and bacteria

**Fig. 1:**
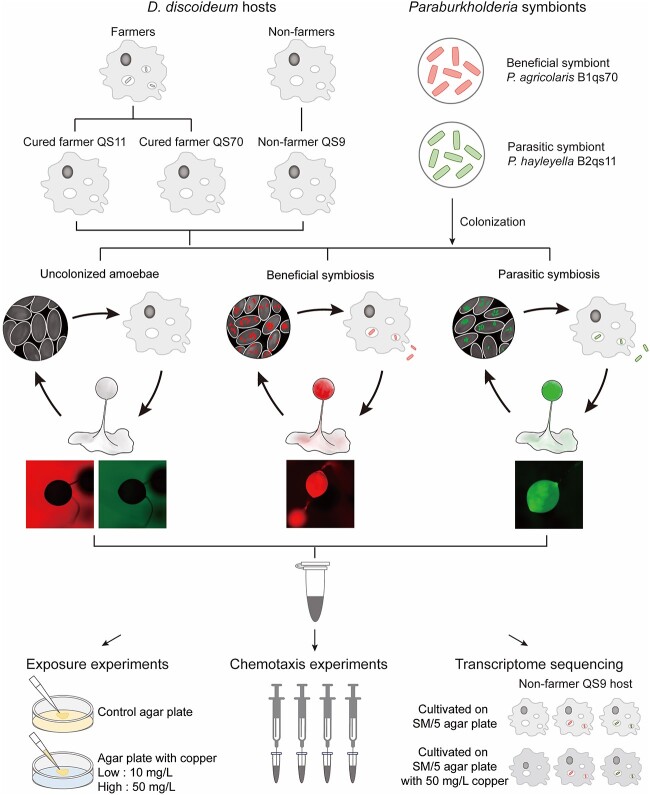
Schematic flow chart of experimental design. The experiment explores the effect of copper stress on amoebae and their associated bacteria. We used three field-collected amoeba hosts and two *Paraburkholderia* symbionts isolated from amoebae to construct six symbiotic relationships with different symbiont types and coevolution history. Both uncolonized and *Paraburkholderia* colonized amoebae were used for exposure experiments to identify the toxicity of excessive copper. We used amoeba supernatant as a chemoattractant and counted the number of bacteria in the syringe using the flat colony counting method to investigate the effect of copper on the chemotaxis of the symbionts. We further analyzed the response mechanism of amoebae to copper stress using transcriptome sequencing.

This study used one *D*. *discoideum* non-farmer strain QS9, which carries no *Paraburkholderia* symbionts in its natural environment, and two farmer strains QS11 and QS70 which are native hosts of the parasitic symbiont *P*. *hayleyella* and the beneficial symbiont *P*. *agricolaris*, respectively [22, 24]. Farmer strains QS11 and QS70 were treated with tetracycline or ampicillin–streptomycin to remove their symbionts [21, 22] (Fig. 1).

We grew *D. discoideum* strains from previously frozen spores on SM/5 agar plates (2.0 g of glucose, 2.0 g of BactoPeptone (Oxoid), 2.0 g of yeast extract (Oxoid), 0.4 g of MgSO_4_·7H_2_O, 1.9 g of KH_2_PO_4_, 1.0 g of K_2_HPO_4_, and 15.0 g of agar per liter) with food bacterium *Klebsiella pneumoniae* KpGe at 21°C. RFP-labeled version of *P. agricolaris* strain B1qs70 and GFP-labeled version of *P. hayleyella* strain B2qs11 were used in this study [23]. All bacteria were cultured on SM/5 agar plates at room temperature.

### Determination of copper content

Minimum inhibitory concentrations (MICs) experiments were performed to determine the susceptibility of three bacteria used in this study to copper and further decide the experimental concentration. *K. pneumoniae* KpGe, *P. agricolaris* B1qs70, and *P. hayleyella* B2qs11 were first revived on SM/5 agar plates at room temperature. Then we picked the monoclone of these bacteria into liquid SM/5 medium, and cultured them at 21°C and 200 rpm to the logarithmic phase. The bacteria suspensions were diluted to 10^−2^ and 50 μl of the diluted solution was added into 150 μl fresh liquid SM/5 medium with different copper contents (0, 10, 30, 40, 50, 60, 70, 90, 100, 110, 130, 150, 200, 250, and 300 mg/L) in 96-well plates and incubated at 21°C. The experimental range of copper concentration was based on the environmental concentration of copper in contaminated soil and the concentration of ecotoxicity [37–39]. OD values were measured at 600 nm to estimate the growth of bacteria after 24 h.

### Determination of bacterial growth curves

The suspensions of *K. pneumoniae* KpGe, *P. agricolaris* B1qs70, and *P. hayleyella* B2qs11 at logarithmic phase were inoculated at an initial ratio of 2% into fresh liquid SM/5 medium with chosen copper contents or without copper at 21°C and cultured at 200 rpm with three replicates. OD values of 200 μl cell suspension were measured at 600 nm over a period of 106 h. Bacteria growth rate was the slope of the linear portion of the growth curve by plotting Ln (OD_600_) as the y-axis and time as the x-axis. Biomass yield refers to the maximum OD_600_ reached by the culture [40, 41].

### Exposure experiments

We plated 2 × 10^5^*D. discoideum* spores with 200 μl of *K. pneumoniae* KpGe suspensions set at an OD_600nm_ of 1.5 on SM/5 agar plates with different copper contents and incubated at 21°C for 5 days. All treatments were done with four replicates. In order to exclude the effect of the food bacterium *K. pneumoniae* KpGe on the growth of *D. discoideum*, we also plated 2 × 10^5^*D. discoideum* spores with 200 μl of *K. pneumoniae* KpGe suspensions set at an OD_600nm_ of 30 on Non-nutrient agar (NNA) plates (2.2 g of KH_2_PO_4_, 1.7 g of K_2_HPO_4_, and 15.0 g of agar per liter) with different copper contents on which *K. pneumoniae* KpGe could not grow, then incubated them at 21°C for 7 days in quintuplicate.

### *Paraburkholderia* colonization

We constructed six symbiotic systems using two different symbiont types (beneficial and parasitic symbionts) and three amoeba strains (native and non-native hosts) to investigate the effects of symbiont type and coevolution histories on the symbiotic response to copper stress. We mixed each *Paraburkholderia* strain suspension with *K. pneumoniae* KpGe suspension (both set at an OD_600nm_ of 1.5) at a 5% *Paraburkholderia*/*K. pneumoniae* KpGe (vol/vol) ratio as indicated. We then plated 2 × 10^5^*D. discoideum* spores with 200 μl of these bacterial mixtures on SM/5 agar plates with different copper contents and incubated them at 21°C for 5 days. To explore the effect of endosymbiotic biomass on amoeba’s fitness under different copper content, we used 1%, 3%, and 5% *Paraburkholderia*/*K. pneumoniae* KpGe (vol/vol) ratio to colonize *D. discoideum* strains. The 2 × 10^5^*D. discoideum* spores with 200 μl of these bacterial mixtures were also cultivated on SM/5 at 21°C for 5 days with three replicates.

### Fitness analysis

Spore productivity was used to measure the amoeba’s fitness. We flooded the plates of the above experiments with 10 ml KK2 (2.25 g of KH_2_PO_4_, 0.67 g of K_2_HPO_4_) containing 0.1% NP-40 and collected the spores into a falcon tube. Spores were counted on a hemocytometer using a light microscope.

### Symbiosis analysis

Fluorescence intensity per 2 × 10^5^*D. discoideum* spores was used to represent the number of symbionts within spores. We picked up the fruiting body sorus using a sterile inoculating loop and collected the spores into a 2 ml falcon tube. We counted spores on a hemocytometer using a light microscope and measured fluorescence intensity using Varioskan LUX Multimode Microplate Reader (Thermo Fisher Scientific, MA, USA). Green fluorescence was tested under an excitation wavelength of 485 nm and an emission wavelength of 515 nm [42], and red fluorescence was tested under an excitation wavelength of 555 nm and an emission wavelength of 585 nm [43]. A fluorescence microscope (Discover Echo Inc., CA, USA) was used to observe the symbiotic relationship between amoebae and bacteria.

### Motility test of bacteria

Motility is the prerequisite for bacterial chemotaxis. We tested the motility of two symbionts on SM/5 plates supplemented with 0.3% agar [44]. We picked the monoclone of these bacteria into liquid SM/5 medium and cultured them at 21°C and 200 rpm to the stationary stage. Then we inoculated 10 μl of bacteria suspensions (set at an OD_600nm_ of 2.0) at the center of the agar plates and stabbed the pipette tip into the agar during inoculation. Food bacteria *K. pneumoniae* KpGe was used as negative control [24]. The plates were cultivated at 21°C for 3 days and the colony diameters were measured. Three replicates were done for each strain.

### Capillary chemotaxis experiments

Supernatants from log-growth amoebae were used in the chemotaxis experiment. We collected cells of three *D. discoideum* strains at the logarithmic phase with KK2 buffer and centrifuged the collected suspension at 1500 g for 3 min to remove the remaining bacteria. The washing process was repeated three to four times. We placed 10^8^ amoeba cells in a 15-ml centrifuge tube containing KK2 buffer and incubated at 21°C for 12 h. After incubation, we isolated the supernatant by centrifuging the amoeba suspension in an Eppendorf Centrifuge 5804R at 1500 g for 3 min and further isolated the supernatants at 12 000 g for 10 min at 4°C. We then filtered the supernatants through a 0.22 μm sterile syringe filter and kept them at 4°C before use.

To investigate the chemotactic responses of *Paraburkholderia* symbionts (*N* = 2) to amoeba supernatants (*N* = 3), we used a capillary assay described before [45]. We added 200 μl of bacteria suspensions (set at an OD_600nm_ of 2.5) into a 1.5-ml centrifuge tube and placed a 1 ml injection syringe containing 100 μl of amoeba supernatant into the centrifuge tube. KK2 buffer was used as blank control. The needle of the syringe was used as the chemotaxis capillary. After 6 h incubation at room temperature, we collected the amoeba supernatants, and viable cells of bacteria were measured using the colony-forming unit method. Three replicates were done for each combination. The accumulation of *Paraburkholderia* in response to the amoeba secretions was expressed in terms of the number of bacteria. The data were log-transformed for data plotting.

### Genomic analysis

The genomes of *P. agricolaris* type strain BaQS159 (GenBank accession number: GCA_009455635.1) and *P. hayleyella* type strain BhQS11 (GenBank accession number: GCA_009455685.1) were downloaded from the NCBI database. We used KofamKOALA [46] (https://www.genome.jp/tools/kofamkoala/) to annotate copper resistance genes in these two species. The comparison was conducted to further explain the different copper tolerances of these two species.

### Transcriptome sequencing and analysis

Transcriptome sequencing and analysis were used to reveal the potential response mechanisms of different symbiont patterns on copper stress. Six groups including the uncolonized QS9 control group, uncolonized QS9 copper-treated group, *P. agricolaris* colonized QS9 control group, *P. agricolaris* colonized QS9 copper-treated group, *P. hayleyella* colonized QS9 control group, and *P. hayleyella* colonized QS9 copper-treated group were used for transcriptome sequencing. It has been previously determined that amoeba cells reached the logarithmic phase at 32–36 h [20]. When cells reached log-phase growth, 5 × 10^6^ amoeba cells were collected from each group with three replicates for transcriptome sequencing. RNA was extracted with TRIzol Reagent (Invitrogen, CA, USA) according to the manufacturer’s instructions, and samples were sequenced at Majorbio Technology Co., Ltd. (Shanghai, China) by Illumina NovaSeq 6000 sequencer (Illumina, CA, USA). Raw paired-end reads were trimmed and quality-controlled by fastp [47] with default parameters. Clean reads were then separately aligned to the *D. discoideum* reference genome (GCF_000004695.1) with orientation mode using HISAT2 software [48]. Differentially expressed genes (DEGs) were identified using the R software package DESeq2 v1.24.0 [49]. The results produced by DESeq2 were expressed as the estimated log2 fold change of the unigene expression level between treatments, with Benjamini−Hochberg adjustment and a false discovery rate of 0.05. Gene Ontology (GO) functional enrichment and Kyoto Encyclopedia of Genes and Genomes (KEGG) pathway analysis were carried out by Goatools [50] and KOBAS [51], respectively.

### Statistical analysis

All analyses were performed and figures were plotted using R version 4.2.1 and GraphPad Prism 9.5.1 software (GraphPad Software Inc., CA, USA). The statistical significance of multiple pairwise comparisons was based on the one-way analysis of variance (ANOVA) followed by Fisher’s LSD post hoc test and Bonferroni adjustment. Permutational multivariate analysis of variance was performed using the Adonis function with 999 permutations and sample ordination was visualized in a principal component analysis (PCA). General linear models (GLMs) were used to analyze the influence of different factors and their interactions on the spore productivity data. A *P* value less than .05 is considered statistically significant.

## Results

### Beneficial symbiont *P. agricolaris* was more resistant to copper stress

**Fig. 2:**
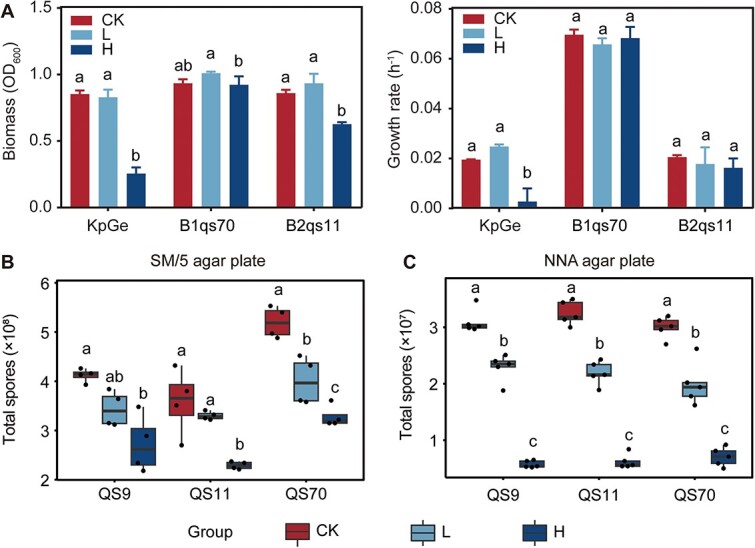
Copper effect on the growth of bacteria and amoebae. (A) the biomass yield and growth rate of food bacteria *K. pneumoniae* and two symbiont bacteria *P. agricolaris* and *P. hayleyella* under different copper concentrations. (B-C) The number of total amoeba spores exposed to different copper concentrations (L: 10 mg/L and H: 50 mg/L) and the control group (CK) counted on (B) nutrient and (C) non-nutrient agar plates. Pairwise comparisons were performed in different treatments of each group. Different lowercase letters represent significant differences between factor levels, based on Fisher’s least significant difference (LSD) test (*P* < 0.05).

In order to ensure the normal growth of food bacteria and endosymbiotic bacteria, we selected two concentrations of copper (low: 10 mg/L and high: 50 mg/L) in this study based on the results of the MICs experiment (See online supplementary material for a colour version of Fig. S1). The control group was cultured with normal SM/5 agar plates. The growth curves of *K. pneumoniae* KpGe, *P. agricolaris* B1qs70, and *P. hayleyella* B2qs11 in the control group and two copper concentration groups were further measured (See online supplementary material for a colour version of Fig. S2). The three bacteria showed different resistance to copper (Fig. 2A). Food bacteria *K. pneumoniae* KpGe maintained normal growth at low copper concentrations (final biomass, *P* = .7121; growth rate, *P* = .1180), but the high concentration of copper significantly decreased its growth rate and final biomass yield (*P* < .001). Unlike food bacteria KpGe, copper had no significant effect on the growth rate of two symbionts (*P. agricolaris*, *P* = .2674; *P. hayleyella*, *P* = .4170). Both symbiont bacteria could grow under high concentrations of copper, but the biomass of *P. hayleyella* B2qs11 was significantly inhibited under high copper concentrations (*P* < .001), indicating that *P. agricolaris* B1qs70 was more tolerant to copper stress.

We further identified different response mechanisms of the two symbionts to copper stress by KEGG annotation. Genes involved in the *cop* system were identified from both *P. agricolaris* and *P. hayleyella* strains. However, relative genes of *cus* system were deficient in *P. hayleyella* type strains (Table S1). The *cus* system participated in periplasmic copper detoxification after CopA extruded the excess copper from the cytoplasm [52], and contributed to the copper tolerance of bacteria [53, 54]. The deficiency of *cus* system may result in the different tolerance of these two endosymbiotic strains to copper stress.

### Copper can directly inhibit *D. discoideum* amoeba’s fitness

The effect of copper stress on amoebae was tested using exposure experiments on SM/5 agar plates. A high concentration of copper significantly inhibited the growth of all three *D. discoideum* strains (Fig. 2B). Only strain QS70 appeared more sensitive to low concentration of copper with its total spore productivity decreasing by 22.9% after copper treatment (Fig. 2B). When cultured on SM/5 agar plates, both copper concentration (*P* < .001, GLM) and amoeba strain (*P* < .001, GLM) were main factors influencing spore productivity, whereas their interactions showed no significant effect on amoeba’s fitness (*P* = .126, GLM). Considering the inhibition of a high concentration of copper on the growth of food bacteria, we later performed exposure experiments on a NNA medium to eliminate the interference of food bacteria (Fig. 2C). All three strains of *D. discoideum* produced fewer spores with increasing copper concentrations. Under non-nutritive conditions, copper concentration (*P* < .001, GLM) was the only factor affecting *D. discoideum*’s fitness. These results indicated that a high concentration of copper decreased amoeba’s fitness by acting directly on amoeba cells.

### Symbiont types determine the response of amoeba–bacteria symbiosis to copper stress

**Fig. 3:**
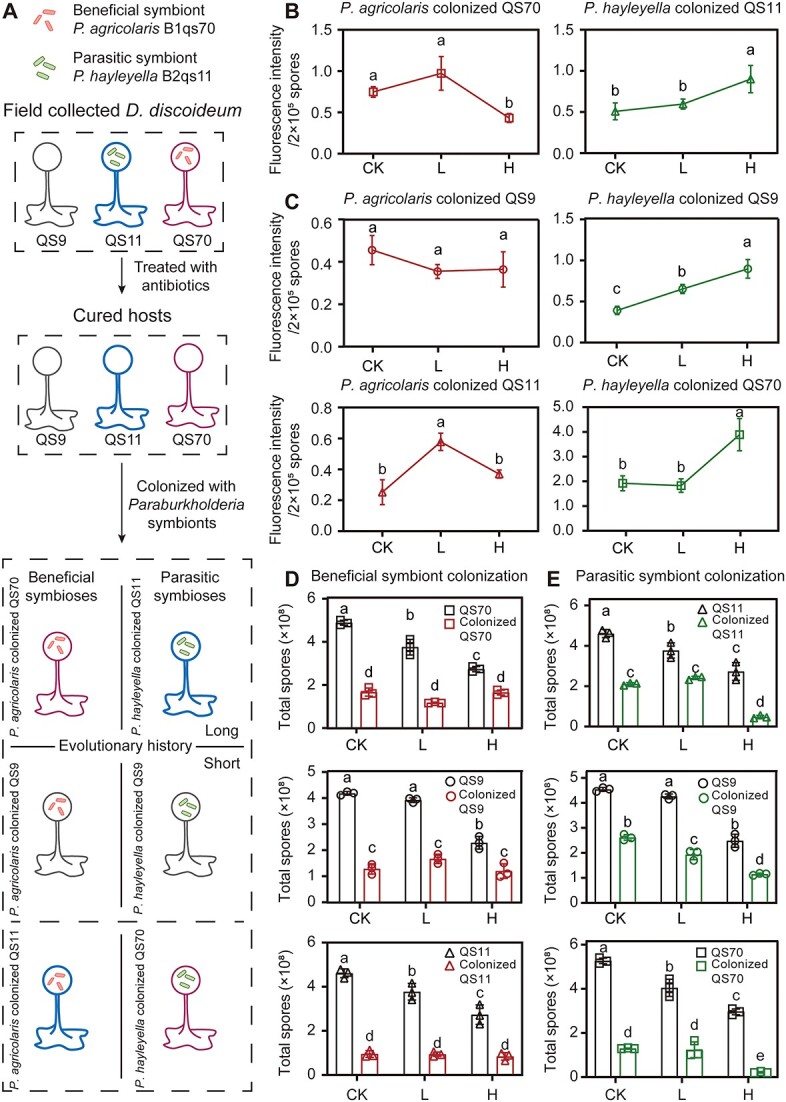
(A) Illustration of amoeba-bacteria symbiotic relationships used throughout the study. Amoeba strains were originally harvested from the wild in three different states: Uncolonized with symbionts, or naturally colonized by *P. agricolaris* or *P. hayleyella*. Strains were treated with antibiotics to eliminate symbionts. Antibiotic-treated strains were subsequently exposed to *Paraburkholderia* to form a symbiotic relationship. Based on the symbiont type, six symbioses can be divided into beneficial symbiosis and parasitic symbiosis. Symbionts with their native hosts had longer coevolution history and the other four symbioses had shorter coevolution history. (B-C) fluorescence intensity changes of *Paraburkholderia* colonized amoebae under copper stress. (B) Symbiotic systems with a longer evolution history, (C) symbiotic systems with a short evolution history. (D-E) comparisons of total spores’ changes among uncolonized amoebae and *Paraburkholderia* colonized amoebae under copper stress. (D) Amoebae colonized by beneficial symbiont *P. agricolaris*, (E) amoebae colonized by parasitic symbiont *P. hayleyella*. Pairwise comparisons were performed in different treatments of each group. Different lowercase letters represent significant differences between factor levels, based on Fisher’s least significant difference (LSD) test (*P* < 0.05).

In this study, we constructed two symbiotic systems with long coevolutionary histories, including *P. agricolaris* colonized QS70 and *P. hayleyella* colonized QS11, as well as four systems with shorter coevolutionary histories, including *P. agricolaris* colonized QS9, *P. agricolaris* colonized QS11, *P. hayleyella* colonized QS9, and *P. hayleyella* colonized QS70 (Fig. 3A). Our findings revealed that the responses of amoeba-bacteria symbioses to copper stress varied depending on the symbiont types. Specifically, a high concentration of copper was observed to decrease the number of beneficial symbionts while increasing the number of parasitic symbionts within the amoeba spores in symbiotic systems with long coevolutionary histories (Fig. 3B and See online supplementary material for a colour version of Fig. S3). Although the colonization of beneficial symbionts was not significantly affected by high copper concentration in symbiotic systems with short coevolutionary histories (host QS9, *P* = .258; host QS11, *P* = .188), the colonization of parasitic symbionts exhibited a similar response pattern to those observed in systems with long evolutionary histories (Fig. 3C). These findings suggest that symbiont type plays a crucial role in determining the response of symbiosis to copper stress. Even though copper stress promotes the colonization of parasitic symbionts in amoeba hosts, its effect on the symbiotic relationship between amoebae and beneficial symbionts is limited.

In many plant-bacteria symbiotic relationships, copper-resistant symbionts facilitated the adaptation of hosts to copper stress [55–57]. Given the presence of copper tolerance in *Paraburkholderia* symbionts, we further examined the effect of colonization on amoeba’s copper resistance. We conducted tests to assess the effect of copper on the fitness of *Paraburkholderia*-colonized amoebae on SM/5 agar plates. The results revealed that the type of symbiont altered the effect of copper on amoeba fitness (Fig. 3D and E). Specifically, the spore productivity of beneficial symbiont-colonized amoebae no longer decreased with increasing copper concentrations (Fig. 3D). Conversely, the fitness of parasitic symbiont-colonized amoebae decreased further with increasing copper concentrations (Fig. 3E). Furthermore, we calculated the inhibition rates of spore productivity caused by symbiont colonization, copper stress, and their combined effect. We found that both single copper stress and symbiont colonization had a significant inhibitory effect on spore productivity (See online supplementary material for a colour version of Fig. S4). However, after colonization by beneficial symbionts, copper stress did not significantly inhibit spore productivity (See online supplementary material for a colour version of Fig. S4A). In contrast, after colonization by parasitic symbionts, copper stress maintained a high inhibitory effect similar to that observed in the uncolonized group (See online supplementary material for a colour version of Fig. S4B).

In order to exclude the effect of symbiont biomass on spore productivity, we used different ratios of *Paraburkholderia* symbionts (1%, 3%, and 5%) to colonize their native *D. discoideum* hosts (See online supplementary material for a colour version of Fig. S5). We found that after colonization of beneficial symbionts, the symbiont biomass (*P* = .629, GLM), copper concentration (*P* = .428, GLM), and their interactions (*P* = .115, GLM) had no significant effect on amoeba’s fitness (Table S2). In contrast, the spore productivity of parasitic colonized amoebae was significantly affected by the symbiont biomass, copper concentration, and their interactions (*P* < .01, GLM) (Table S2). These results suggest that colonization by beneficial symbionts enhances the resistance of amoeba hosts to copper stress. Furthermore, copper stress promotes a shift in the symbiotic relationship between amoebae and beneficial symbionts in favor of the amoeba hosts, and also enhancing the pathogenic effects of parasitic symbionts on hosts.

### Copper stress altered the chemotaxis of symbionts toward amoeba hosts

**Fig. 4:**
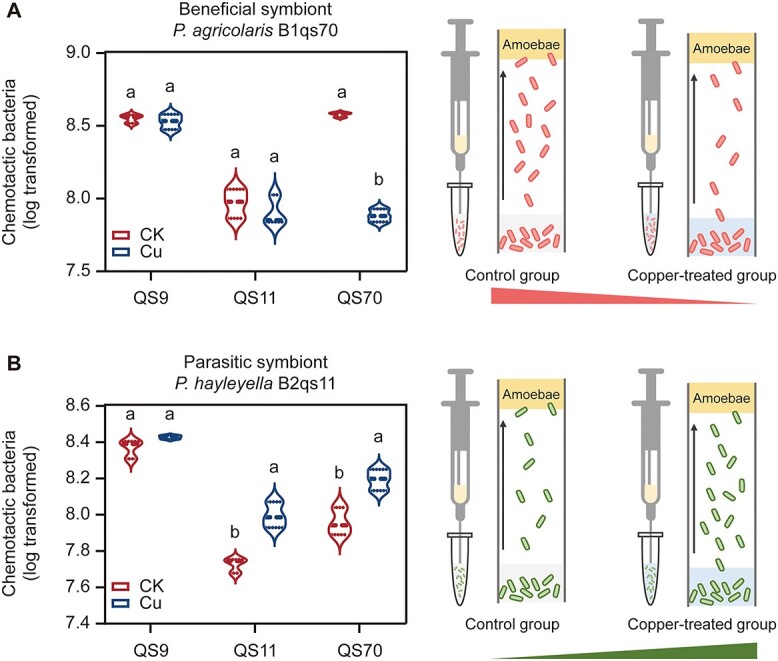
Chemotaxis of two symbiont bacteria to supernatants of amoeba hosts in the control group and high copper treated group. Pairwise comparisons were performed in different treatments of each group. Different lowercase letters represent significant differences between factor levels, based on Fisher’s least significant difference (LSD) test (*P* < 0.05).

In addition to amoeba phagocytosis, the chemotaxis of symbionts can also influence the formation of amoeba–bacteria symbiotic relationships. We investigated the effect of copper stress on the motility of two symbionts by using a standard plate-based bacterial swimming assay, with non-motile *K. pneumoniae* as a negative control (See online supplementary material for a colour version of Fig. S6). Results showed that a high concentration of copper had no significant effect on the motility of two symbionts (*P. agricolaris*, *P* = .860; *P. hayleyella*, *P* = .926; See online supplementary material for a colour version of Fig. S6). We further used amoeba supernatant as inducers to test the effect of high concentrations of copper on bacterial chemotaxis (Fig. 4). We found that copper stress showed opposite effects on the chemotaxis of two symbiont types. For the beneficial symbiont *P. agricolaris* B1qs70, copper stress showed no significant effect on its chemotaxis to supernatants of host QS9 and QS11, but significantly reduced its chemotaxis when the supernatant of QS70 was used as an inducer (Fig. 4A). Conversely, the chemotaxis of parasitic symbiont *P. hayleyella* B2qs11 to amoeba supernatants increased in response to copper stress (Fig. 4B). These findings revealed that copper stress altered the chemotaxis of symbionts to amoeba hosts, and affected the formation of symbiotic relationships by mediating the dynamic behavior of both amoeba hosts and their symbionts.

### Copper stress significantly changes the transcriptome profiles of amoebae

We performed transcriptome analyses to explore the molecular mechanisms of amoebae and amoeba-bacteria symbiotic systems in response to copper stress, which produced about 45 million clean reads per sample (Table S3). PCA was used to compare the overall transcriptome expression profile of soil amoebae under different treatments (See online supplementary material for a colour version of Fig. S7A). Results showed that the high copper treatment (50 mg/L) significantly changed amoeba’s transcriptome (*R*^2^ = 0.29, *P* < .01), as indicated by PC1. The correlation analysis further confirmed these patterns (See online supplementary material for a colour version of Fig. S7B).

### Copper serves as a trigger inducing early entry into the developmental stage of *D. discoideum*

**Fig. 5:**
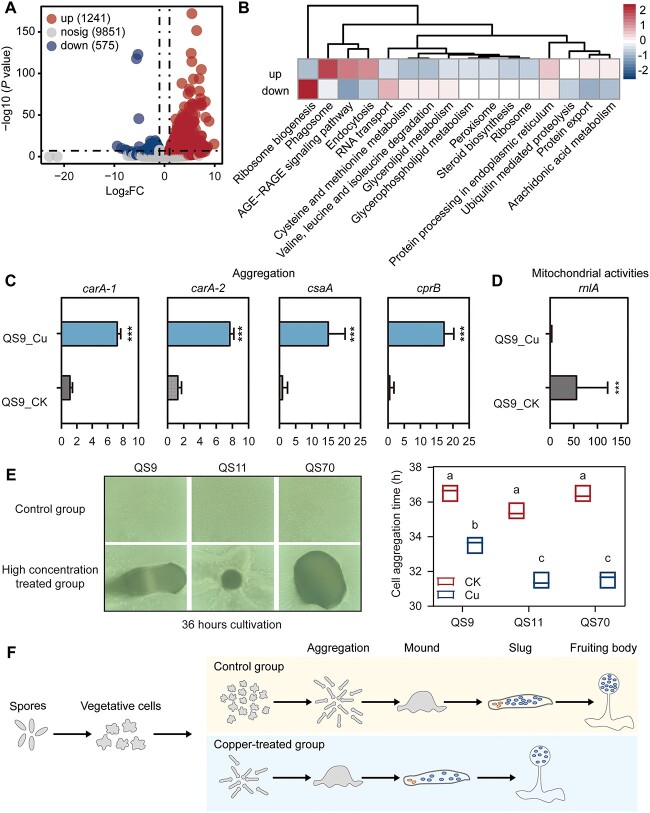
(A) Volcano plot of DEGs in uncolonized amoebae induced by copper stress. (B) Heatmap of gene expressions assigned to KEGG pathways. Gene numbers assigned to each KEGG pathway were normalized. Gene expressions of representative genes related to (C) development and (D) mitochondrial activity. (E) Microscopic morphology of amoeba cells in the control group and high concentration copper treated group after 36 hours of cultivation and the cell aggregation time of each group. (F) Schematic diagram of amoeba inhibition by copper.

To investigate the response mechanism of *D. discoideum* to copper stress, we identified the differentially expressed genes (DEGs) between the uncolonized QS9_CK group and the uncolonized QS9_Cu group. Overall, 1241 DEGs were up-regulated and 575 DEGs were down-regulated under copper stress (See online supplementary material for a colour version of Fig. 5A). To better understand the function of these DEGs, we performed KEGG pathway annotation analysis. Results showed that most up-regulated genes were related to the phagosome, AGE-RAGE signaling pathway, and endocytosis, whereas down-regulated genes were mainly related to ribosome biogenesis and RNA transport pathways (Fig. 5B). The AGE-RAGE signaling pathway is a complex signaling cascade that has been well-studied in many different disease states [58, 59]. However, this signaling could also elicit the activation of multiple intracellular signal pathways including NADPH oxidase, calcium, and MAPKs [60, 61]. Results of GO enrichment analysis showed that genes involved in response to starvation and nutrient levels, cell development, and cell communications were enriched under copper stress (See online supplementary material for a colour version of Fig. S8), which indicated that copper might alter the signaling between amoeba cells.

We further focused on DEGs related to the pathways mentioned above and DEGs that play important roles in cell–cell signaling during the *D. discoideum* life cycle. We found that the markers of starvation-induced cAMP signaling and spore formation, including *carA1*, *carA2*, *csaA*, *cprB* [62, 63], and other genes that were essential for post-aggregative development and cell-type specialization were highly expressed under copper stress (Fig. 5C and Table S4). In contrast, genes related to mitochondrial functions, namely *rnlA*, *cluA*, and *atp1*, were significantly down-regulated (Fig. 5D and Table S4). Previous studies have indicated that decreased mitochondrial metabolism drives aggregation and multicellularity of *D. discoideum*, and is accompanied by an increase in cellular and mitochondrial reactive oxygen species [63]. Consistent with these findings, we found that the expression of the *noxB* gene, which encodes NADPH oxidase, was up-regulated with the addition of copper (Table S4).

Mitochondrial inhibition could activate the AMPK–mTORC1 signaling pathway, which regulates growth/proliferation of amoeba cells [64]. Therefore, the activation of developmental signaling pathways might be the reason leading to the decline of amoeba’s fitness. To validate this hypothesis, we conducted observations on the cell morphology of three amoeba strains cultured in both a control group and a high-concentration copper-treated group every 12 hours. Each group underwent three replicates. After 12 hours of cultivation, amoeba spores hatched into vegetative cells in both groups and underwent binary division over the following 12 hours (See online supplementary material for a colour version of Fig. S9A). However, after 36 hours of cultivation, amoeba cells in the copper-treated group entered the developmental stage, whereas the control cells remained in the growth stage. Additionally, the time of cell aggregation in the copper-treated group occurred significantly earlier than in the control group (Fig. 5E). Macroscopic morphology observations of amoebae on the agar plates cultured for 36 hours also supported this growth state as a population phenomenon (See online supplementary material for a colour version of Fig. S9B). Consequently, we concluded that excessive copper inhibited the spore productivity of uncolonized *D. discoideum* by inducing amoeba cells to prematurely enter the developmental stage (Fig. 5F).

### Copper stress induced complex interactions between amoebae and symbionts

**Fig. 6:**
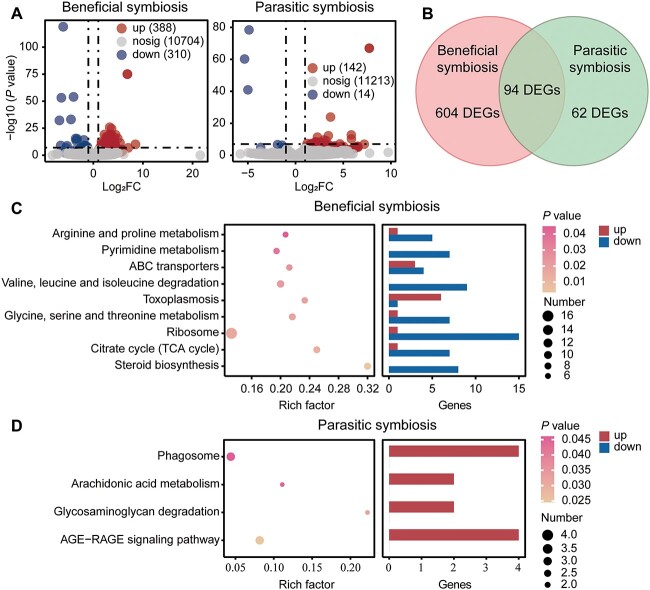
(A) Volcano plot of DEGs in amoebae induced by copper stress in beneficial and parasitic symbiosis. (B) Venn diagram comparing DEGs in amoebae under beneficial and parasitic symbiosis. Values indicate the number of mutual and unique genes between groups. Functions and regulations of DEGs enriched in (C) beneficial symbiosis and (D) parasitic symbiosis.

To investigate the differential effect of copper on *Paraburkholderia* colonized amoebae, we first identified DEGs between *P. agricolaris* colonized QS9 control group and *P. agricolaris* colonized QS9 copper-treated group, and between *P. hayleyella* colonized QS9 control group and *P. hayleyella* colonized QS9 copper-treated group, respectively. The results showed that high concentration of copper led to the up-regulation of 388 genes and down-regulation of 310 genes in *P. agricolaris* colonized amoebae, but only 142 up-regulated genes and 14 down-regulated genes in *P. hayleyella* colonized amoebae (Fig. 6A). The Venn analysis results showed that only 94 DEGs were shared between these two groups, which proved that different types of symbionts resulted in discrepant response mechanisms of amoebae to copper stress (Fig. 6B).

KEGG pathway annotation and enrichment analysis were performed to explore the functions of DEGs. The most enriched pathways of *P. agricolaris* colonized amoebae belonged to nutrient and energy metabolisms, including metabolisms of arginine and proline, pyrimidine, glycine, serine, threonine, and biosynthesis of steroid and citrate cycle. Most of the genes related to these pathways were down-regulated suggesting that nutrient and energy metabolisms were inhibited in *P. agricolaris* colonized amoebae under copper stress (Fig. 6C). In comparison, pathways including AGE-RAGE signaling pathway, phagosome, arachidonic acid metabolism, and glycosaminoglycan degradation were enriched and up-regulated in *P. hayleyella* colonized amoebae (Fig. 6D), indicating that copper stress improved the phagocytosis and cell signaling of *P. hayleyella* colonized amoebae. However, genes involved in response to starvation and nutrient levels, cell development, and mitochondrial activities, which were high-expressed and significantly regulated in uncolonized amoebae, showed no significant change in both *P. agricolaris* colonized amoebae and *P. hayleyella* colonized amoebae (Table S5). These changes suggested that both beneficial and parasitic symbionts ensured the normal growth of amoebae under copper stress and reduced the toxicity of copper to amoebae. Therefore, a further decrease in spore productivity of parasitic colonized amoebae under copper stress might be due to the elevated number of symbiont bacteria.

**Fig. 7:**
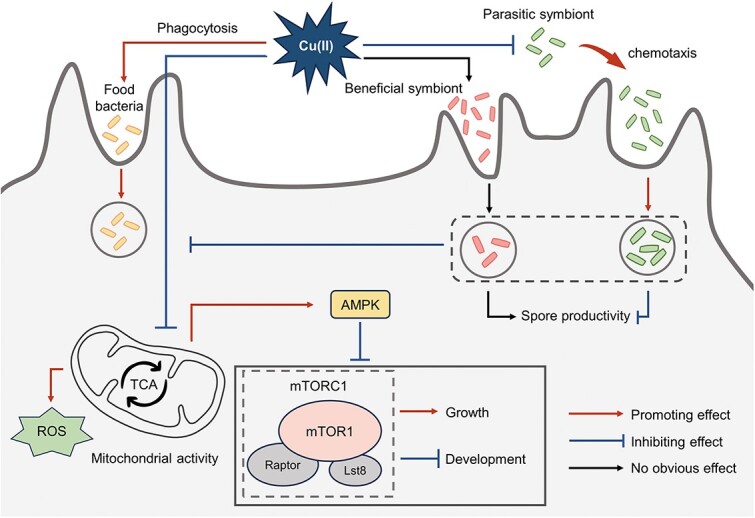
A conceptual model summarizing the effect of copper stress on amoebae and amoeba-bacteria symbiotic relationships.

This study investigated the molecular mechanisms underlying the response of amoebae and amoeba-bacteria symbiotic relationships to copper stress (Fig. 7). We found that copper stress activated the AMPK–mTORC1 signaling pathway by inhibiting mitochondria activity, thereby reducing amoeba’s fitness. However, copper stress promoted the phagocytosis of amoebae. Additionally, *P. agricolaris* had a high tolerance to copper, but *P. hayleyella* was sensitive to high concentrations of copper. We suggested that copper stress might induce a chemotactic response of *P. hayleyella* and prompt bacteria to actively find amoeba hosts. The symbiosis between amoebae and beneficial symbionts was relatively stable, and copper stress no longer affected the amoeba’s fitness after beneficial symbiont colonization. On the contrary, high concentrations of copper enhanced the colonization of parasitic symbionts and further inhibited amoeba’s growth through the toxic effect of parasitic symbionts.

## Discussion

Copper is an essential element in protistan predation that helps kill bacteria, and such predation mechanisms have been proven to induce copper resistance in bacteria [28, 65]. Given the widespread occurrence of copper pollution in soil environments [37, 66], it is especially important to understand how soil amoebae and amoeba-bacteria symbiotic relationships respond to copper stress to block the spread of pathogenic bacteria and maintain the biosafety of soil environments. The study found that copper was a new trigger for developmental cell fate switching of amoebae, and high concentration of copper significantly inhibited amoeba’s growth. In addition, copper stress induced the complex interactions between amoebae and their associated bacteria. Specifically, high concentrations of copper had less impact on the relationship between amoebae and beneficial symbionts but significantly enhanced the formation of a symbiotic relationship between amoebae and parasitic symbionts. Additionally, colonization with beneficial symbionts increased the copper resistance of amoebae, indicating a shift in the symbiotic relationship between amoebae and beneficial symbionts in favor of the amoeba hosts under copper stress. However, copper stress further increased the pathogenic effects of parasitic symbionts on hosts. This study provides new insights into copper toxicity on soil amoebae and potential mechanisms of interactions between amoebae and symbiotic bacteria under environmental stress.

Copper is an essential nutrient implicated in multiple cell proliferation and death pathways [67]. Cuproptosis is a new form of copper-dependent cell death that is highly correlated with mitochondrial respiration [68]. Research on the mechanisms through which cuproptosis is inhibited among normal cells will help us further explore the relationship between cuproptosis and human diseases, especially tumors [69]. Soil amoeba *D. discoideum* is a valuable model for investigating human pathology due to its similarities with mammalian cells and unique developmental life cycle [70, 71]. In the normal life cycle of amoebae, starvation is the major trigger for entry into aggregative development [72]. The growth-to-development transition represses genes encoding ribosomal proteins and induces genes necessary for cAMP [73]. Here, we detected the upregulated expressions of genes *carA1*, *carA2*, *csaA*, *cprB*, and other genes that participate in the developmental stage of amoebae. This suggests that a high concentration of copper induced amoeba cells to prematurely enter the developmental stage. Specifically, copper stress inhibited the binary division of amoebae, resulting in a decrease in the number of amoeba cells and ultimately reduced the spore productivity of amoebae. The multi-protein complexes mTORC1 is important in regulating cell growth/proliferation [48]. Starvation and rapamycin (targeted inhibitors of mTORC1) promote an increase in AMP/ATP ratios to activate AMPK, which further inactivates mTORC1 [64, 74]. We found that copper stress induced changes in transcriptional profile similar to starvation and rapamycin [75], suggesting that copper plays a role in manipulating the activities of AMPK–mTORC1 signaling pathway.

Bacteria can form symbiotic relationships with multiple species, and these symbioses play a fundamental role in ecosystem functioning, host health, and the evolution of biological complexity [76, 77]. Phagocytosis of amoebae is a prerequisite for the formation of a symbiotic relationship between amoebae and bacteria. However, the interaction between bacteria and host is bidirectional. In addition to the uptake by the hosts, bacteria can also actively find and colonize their hosts through motility and chemotaxis [24, 78]. Besides, bacteria can maintain their fitness in stressful environments by triggering a negative chemotactic response [79, 80]. In this study, copper affected the formation of symbiotic relationships by mediating the dynamic behavior of both amoeba hosts and their symbionts. We found that copper stress altered the chemotaxis of symbionts to amoeba hosts. The chemotaxis of *P. hayleyella* towards amoeba hosts was facilitated under copper stress, indicating that environmental stress may prompt *P. hayleyella* to seek amoeba hosts initiatively. Under the complex interactions, the symbiotic relationship between amoebae and beneficial symbionts remained stable, whereas the formation of the symbiotic relationship between amoebae and parasitic symbionts was promoted by copper stress. This study reveals that amoebae serve as natural shelters to help their symbiont bacteria resist environmental stress, which is consistent with our previous research [14].

Although *Paraburkholderia* colonization induces bacterial food carriage of amoeba hosts and sometimes increases spore productivity in food-scarce conditions, these symbionts especially *P. hayleyella*, are harmful to amoeba’s fitness in food-rich conditions [21, 23]. Based on the diverse infection phenotypes and history of symbiosis, *P. hayleyella* and *P. agricolaris* are considered parasitic and beneficial symbionts, respectively. However, the reasons for the differential effects of *Paraburkholderia* clades on amoeba hosts remain unknown. One possible explanation is that each clade of *Paraburkholderia* represents its own symbiotic interaction (neutral, harmful, or beneficial) with the amoebae. Additionally, this difference can also result from the transition of symbiotic relationships from mutualism to parasitism [6]. Results of the comparative genome display that the genome size of *P. hayleyella* was less than half of *P. agricolaris*’s genome. This reduction may force the bacteria to depend more on their host to survive [35]. In this study, copper stress reinforced the pathogenic effects of parasitic symbionts on hosts by significantly increasing the biomass of symbionts within spores. However, the colonization of beneficial symbionts increased the resistance of hosts to copper stress. This symbiont-mediated protection in hosts is widely found in nature [81–83]. These findings provide evidence for the dynamic equilibrium between amoebae and *Paraburkholderia* symbionts, whereas the interaction between amoebae and *Paraburkholderia* symbionts needs to be further explored.

## Supplementary Material

Supplementary_Information_final_wrae100

## Data Availability

The raw sequences used in this study were deposited to the NCBI SRA and can be found under the accession number PRJNA1046722.
