# Supplementary material for: Copper stress shapes the dynamic behavior of amoebae and their associated bacteria
Source: ISME J. 2024 Jun 7;18(1):wrae100. doi: 10.1093/ismejo/wrae100 (PMC11197307; doi:10.1093/ismejo/wrae100)
Supplement: Supplementary_Information_final_wrae100 [file supplementary_information_final_wrae100.docx]

*Supplementary Information for*

**Copper stress shapes the dynamic behavior of amoebae and their associated bacteria**

Yijing Shi^1#^, Lu Ma^2#^, Min Zhou^2^, Zhili He^2^, Yuanchen Zhao^2^, Junyue Hong^2^, Xinyue Zou^2^, Lin Zhang^2^, Longfei Shu^2^*

^1^SCNU Environmental Research Institute, School of Environment, Guangdong Provincial Key Laboratory of Chemical Pollution and Environmental Safety & MOE Key Laboratory of Theoretical Chemistry of Environment, South China Normal University, Guangzhou 510006, China

^2^School of Environmental Science and Engineering, Southern Marine Science and Engineering Guangdong Laboratory (Zhuhai), Guangdong Provincial Key Laboratory of Environmental Pollution Control and Remediation Technology, State Key Laboratory for Biocontrol, Sun Yat-sen University, Guangzhou 510006, China

^#^These authors contributed equally to this work.

***Correspondence**

Prof. Longfei Shu ([shulf@mail.sysu.edu.cn](mailto:shulf@mail.sysu.edu.cn))

School of Environmental Science and Engineering, Southern Marine Science and Engineering Guangdong Laboratory (Zhuhai), Guangdong Provincial Key Laboratory of Environmental Pollution Control and Remediation Technology, Sun Yat-sen University, Guangzhou 510006, China


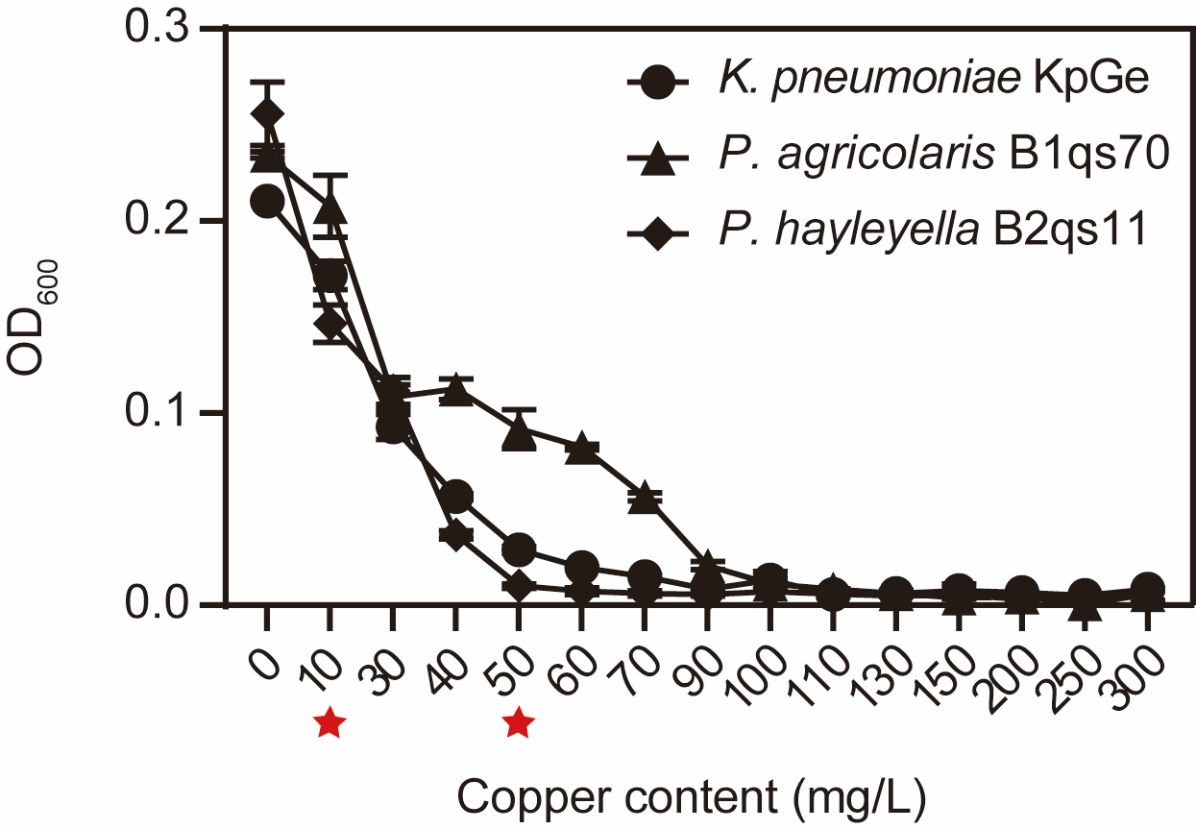


**Figure S1** Minimum inhibitory concentrations (MICs) experiment tested on food bacteria *K. pneumoniae* and two symbiont bacteria *P. agricolaris* and *P. hayleyella*. The red asterisk indicates the concentrations chosen for subsequent experiments.

**
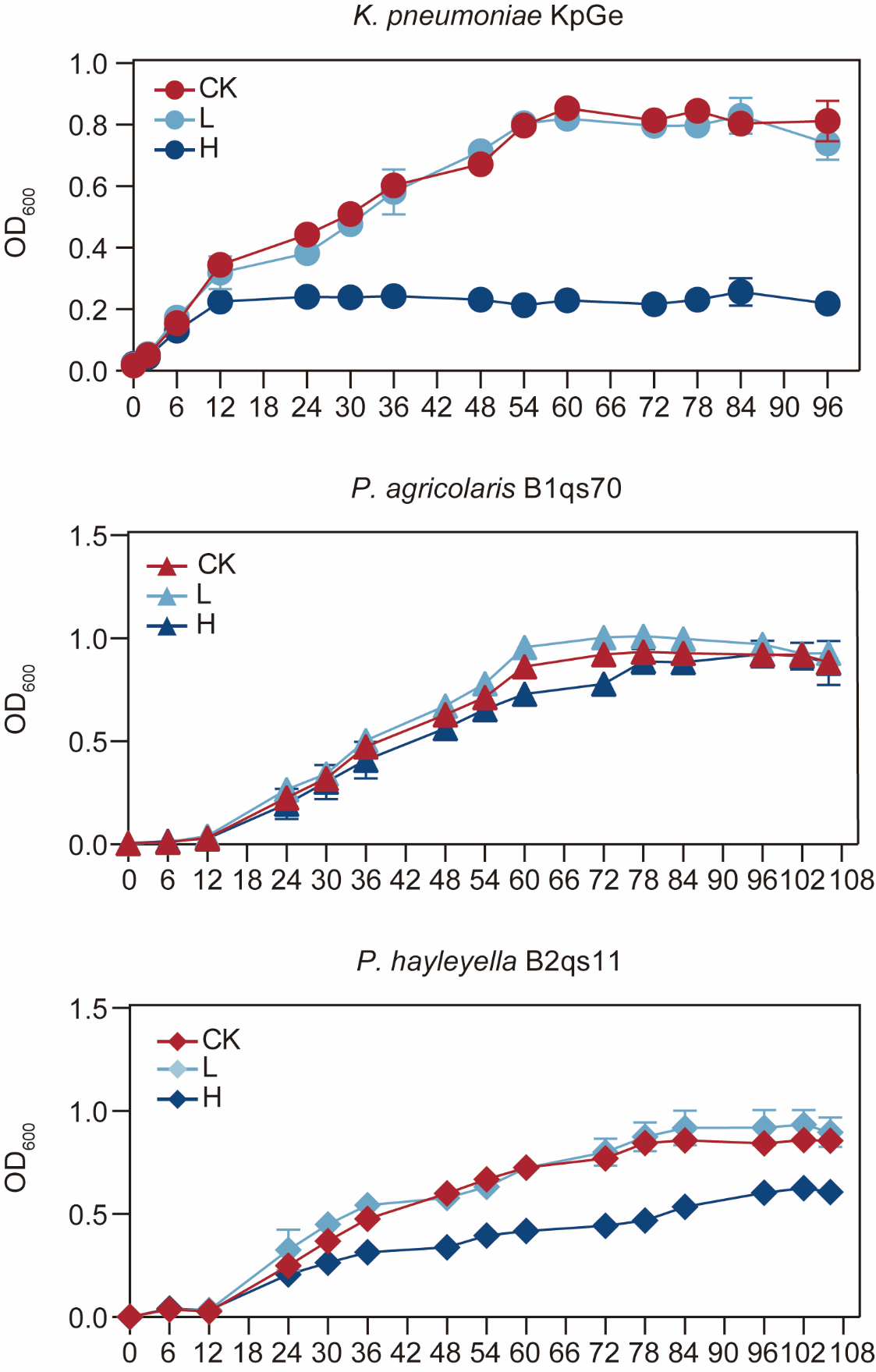
**

**Figure S2** Growth curves of food bacteria *K. pneumoniae* and two symbiont bacteria *P. agricolaris* and *P. hayleyella* under different copper concentrations.


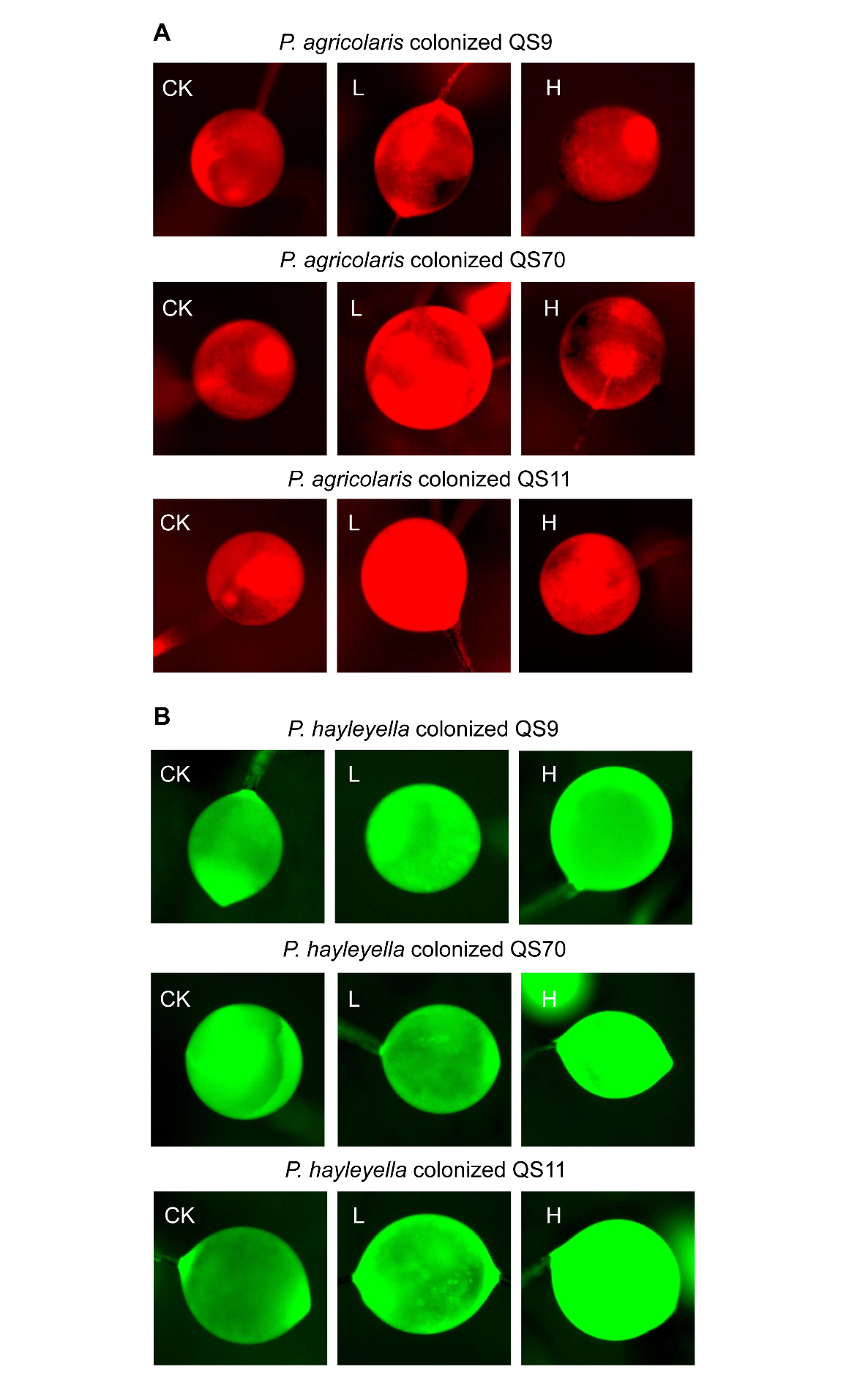


**Figure S3**. Fluorescence photograph of *D. discoideum* clones colonized by *P. agricolaris* and *P. hayleyella* cultured at different copper concentrations.

**
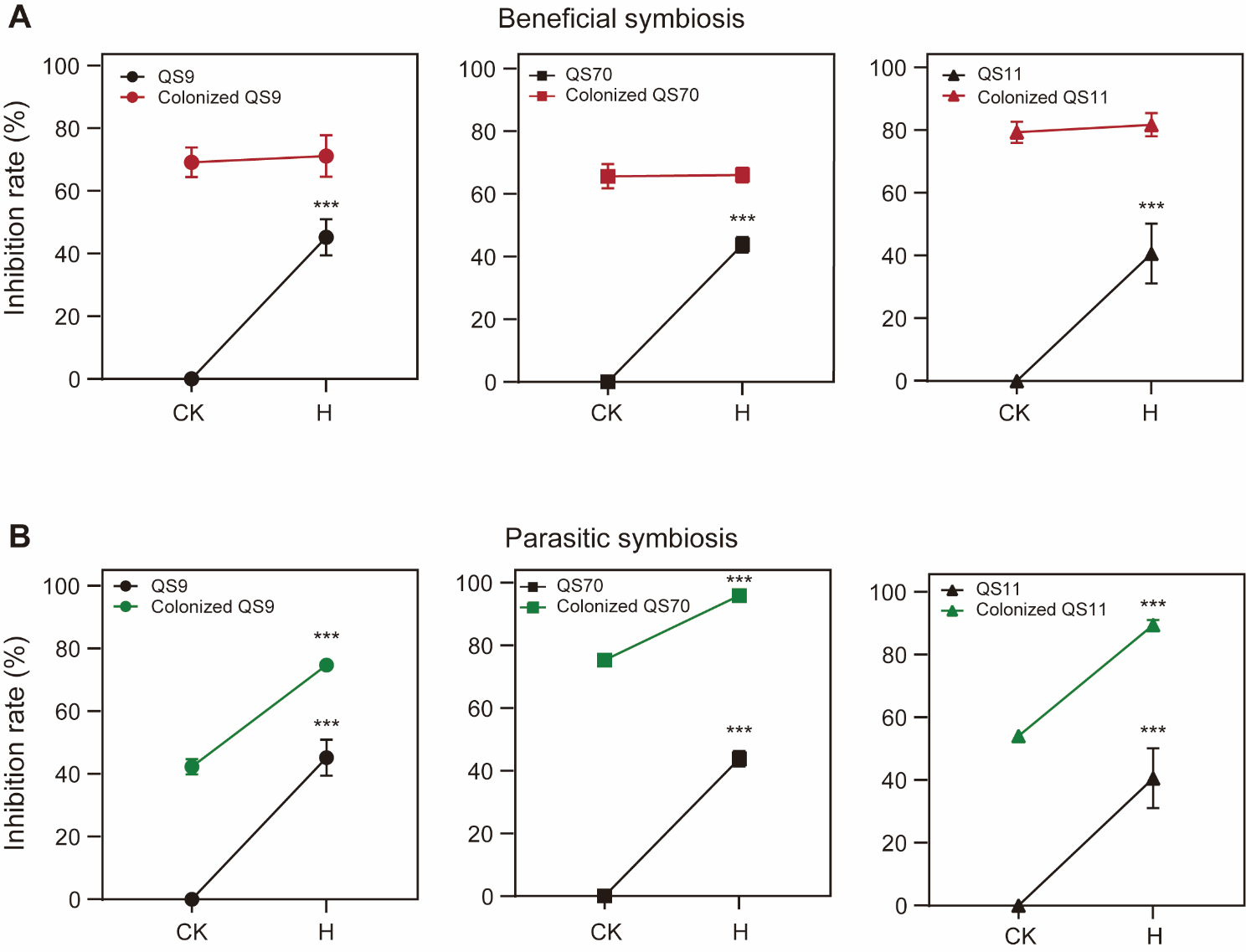
**

**Figure S4**. Inhibition rate of amoebae productivity under symbiont colonization and copper stress. Pairwise comparisons were performed in different treatments of each group. Different lowercase letters represent significant differences between factor levels, based on Fisher’s Least Significant Difference (LSD) test (*P* < 0.05).


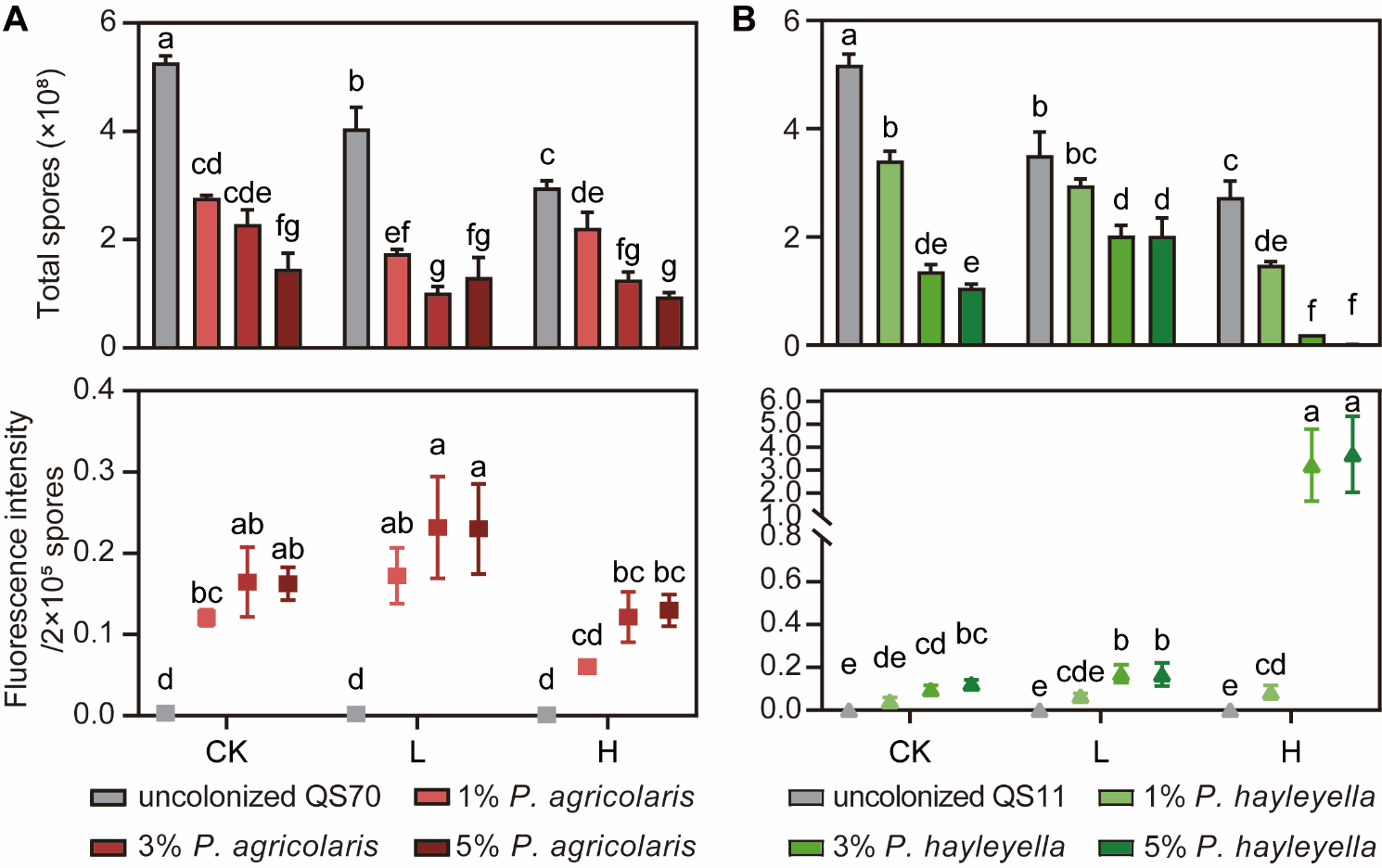


**Figure S5**. Changes in total spores and fluorescence intensity of (A) beneficial symbiosis and (B) parasitic symbiosis under different copper concentrations with different inoculum ratios. Pairwise comparisons were performed in different treatments of each group. Different lowercase letters represent significant differences between factor levels, based on Fisher’s Least Significant Difference (LSD) test (*P* < 0.05).

**
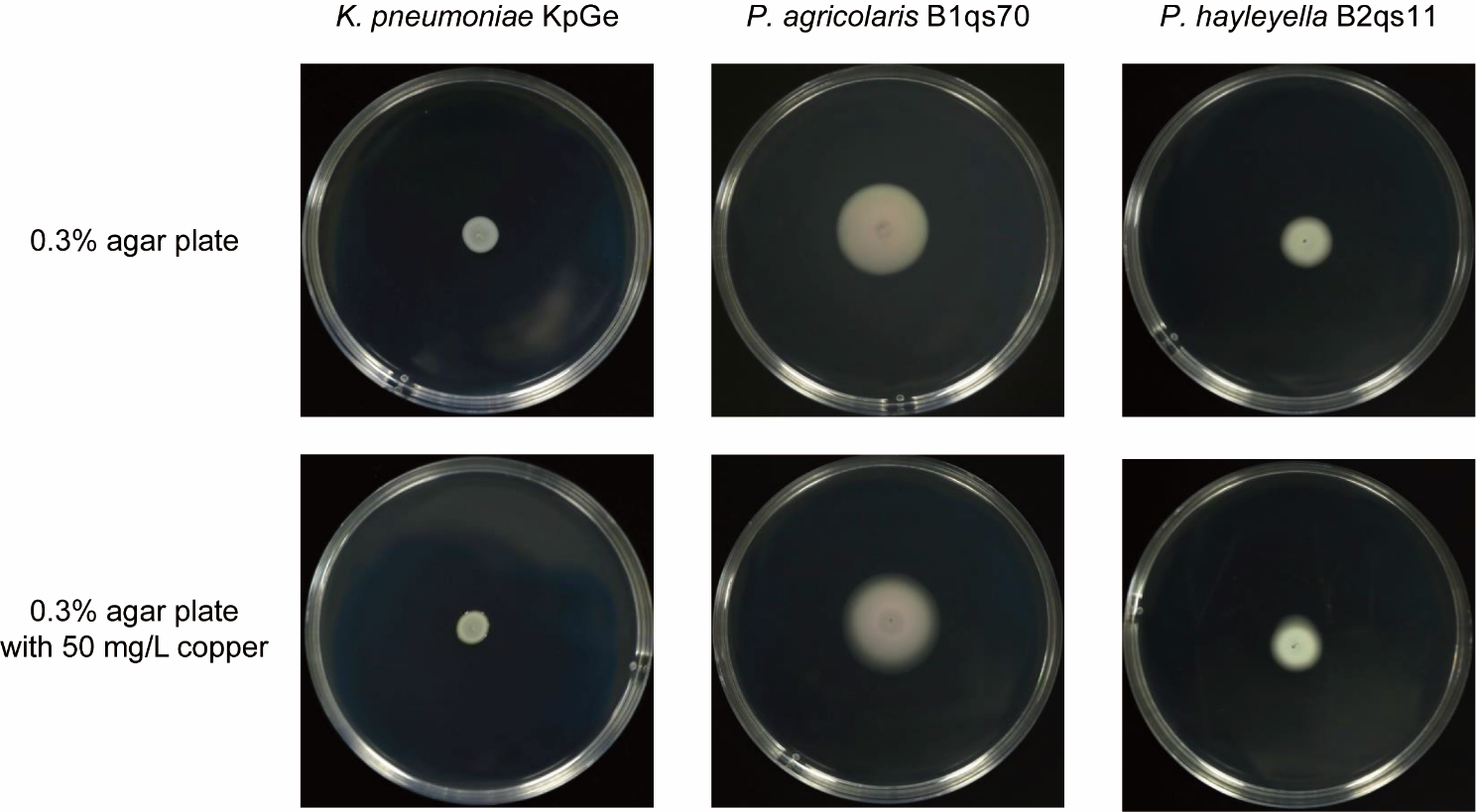
**

**Figure S6**. Swimming motility of *Paraburkholderia* motility. Uncolonized agar is black and bacterial biomass is white. Swimming motility is measured by the diameter of the bacterial colony.


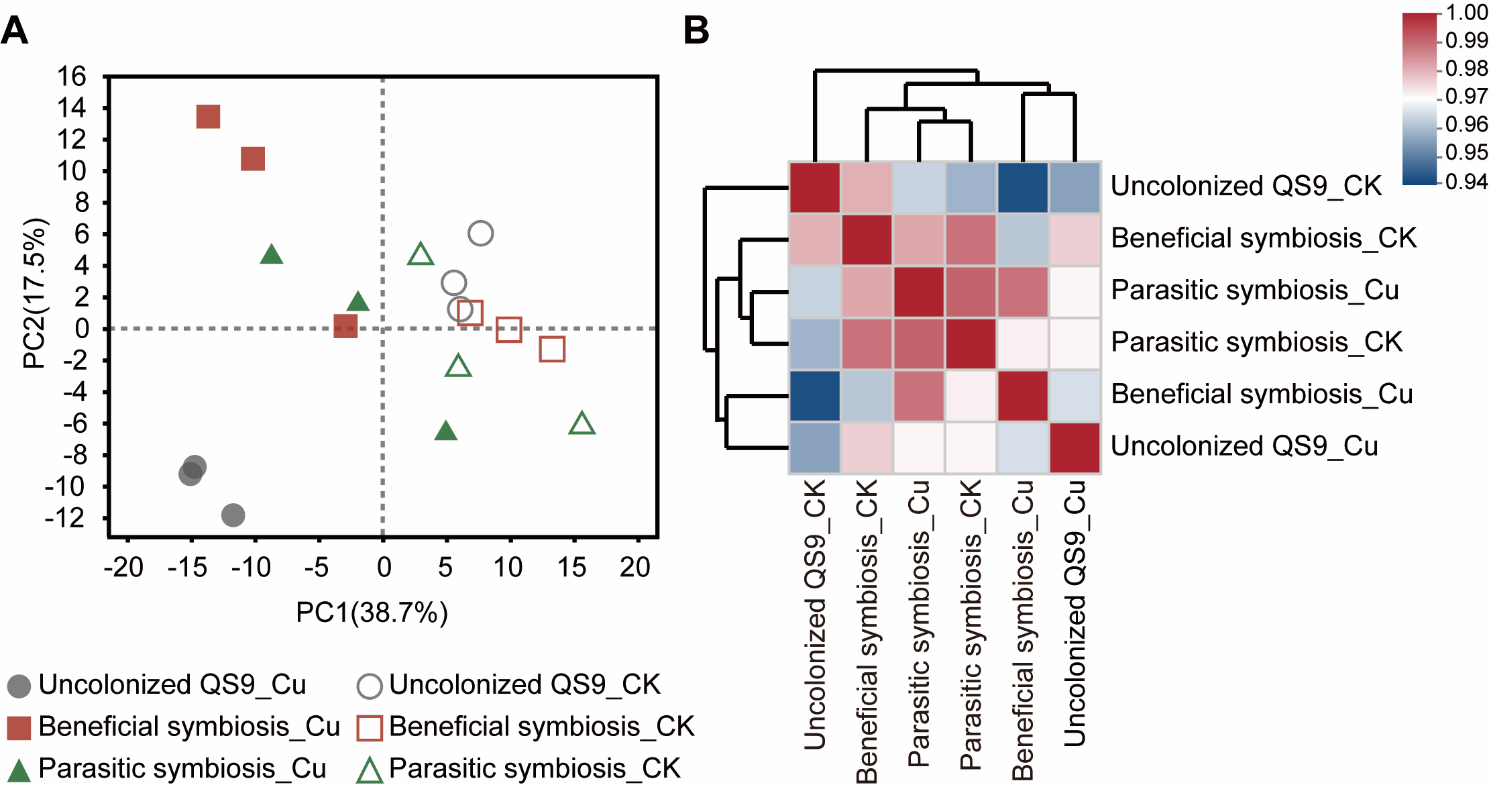


**Figure S7**. (A) Principal component analysis (PCA) plot of six sample groups. (B) Correlation analysis of six sample groups. The color represents the level of correlation (TPM), with red indicating high correlations and blue indicating low correlations.

**
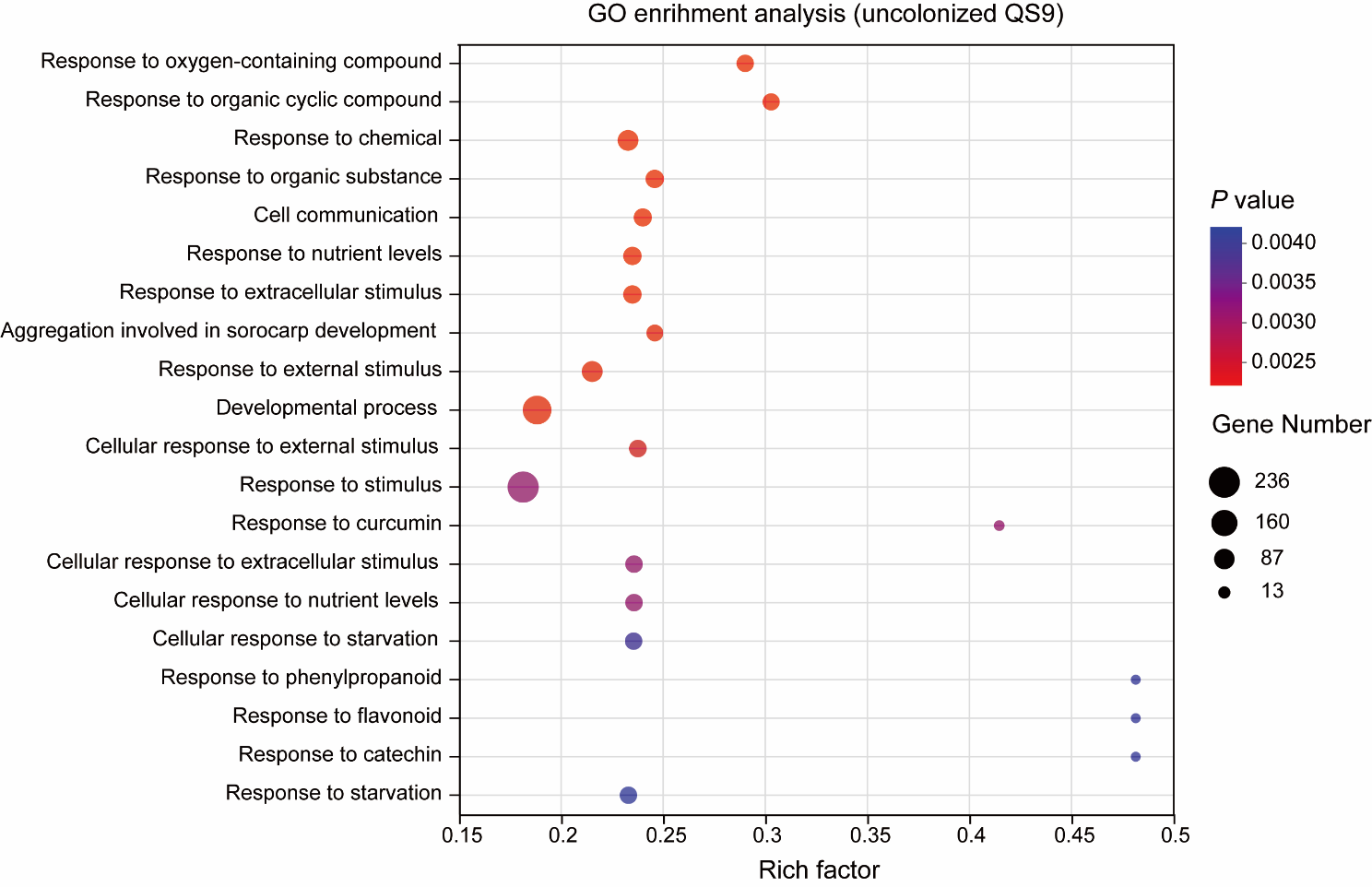
**

**Figure S8.** GO enrichment analysis of differentially expressed genes (DEGs) in uncolonized amoeba induced by copper stress. The size of the dots indicates the number of genes in the GO term, and the color of the dot corresponds to different *p*-adjust values.


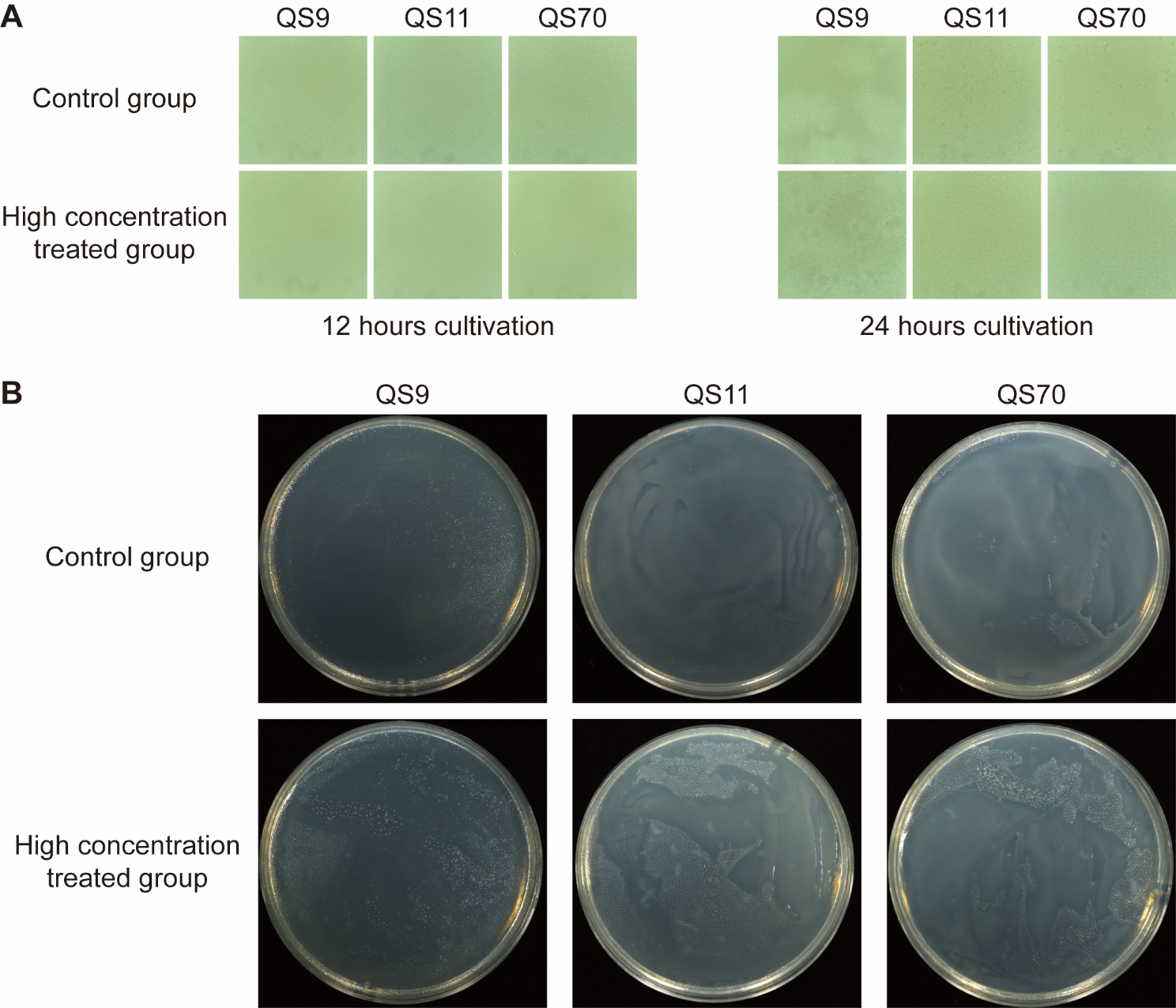


**Figure S9.** (A) Microscopic morphology of amoebae in the control group and high concentration copper treated group every 12 hours during cultivation. (B) Macroscopic morphology of amoebae in the control group and copper-treated group after 36 hours of cultivation.

**Table S1.** Copper resistance genes identified in typical strains of *P. agricolaris* and *P. hayleyella*.

| **KO** | ***P.* *agricolaris*** | ***P. hayleyella*** | **Description** |
| --- | --- | --- | --- |
| K19591 | possess | possess | *cueR*; MerR family transcriptional regulator, copper efflux regulator |
| K17686 | possess | possess | *copA*, *ctpA*, *ATP7*; P-type Cu+ transporter |
| K01533 | deletion | possess | *copB*; P-type Cu2+ transporter |
| K07156 | possess | possess | *copC*, *pcoC*; copper resistance protein C |
| K07787 | possess | deletion | *cusA*, *silA*; copper/silver efflux system protein |
| K07798 | possess | deletion | *cusB*, *silB*; membrane fusion protein, copper/silver efflux system |
| K07810 | possess | deletion | *cusF*; Cu(I)/Ag(I) efflux system periplasmic protein CusF |
| K07665 | possess | deletion | *cusR*, *copR*, *silR*; two-component system, OmpR family, copper resistance phosphate regulon response regulator CusR |
| K07644 | possess | deletion | *cusS*, *copS*, *silS*; two-component system, OmpR family, heavy metal sensor histidine kinase CusS |
| K06201 | possess | possess | *cutC*; copper homeostasis protein |
| K15725 | possess | deletion | *czcC*, *cusC*, *cnrC*; outer membrane protein, heavy metal efflux system |
| K18139 | possess | possess | *oprM*, *emhC*, *ttgC*, *cusC*, *adeK*, *smeF*, *mtrE*, *cmeC*, *gesC*; outer membrane protein, multidrug efflux system |

**Table S2.** The effects of symbiont biomass, copper concentration, and their interactive effects on the spore productivity of amoebae.

|  | **Symbiont biomass** | | **Copper concentration** | | **Biomass × Concentration** | |
| --- | --- | --- | --- | --- | --- | --- |
|  | ***F* value** | ***P* value** | ***F* value** | ***P* value** | ***F* value** | ***P* value** |
| ***P. agricolaris*** | 0.2 | 0.629 | 0.7 | 0.428 | 2.7 | 0.115 |
|  |  |  |  |  |  |  |
| ***P. hayleyella*** | 9.1 | **0.006** | 12.1 | **0.002** | 8.6 | **0.008** |

**Table S3.** Sequencing statistics of the transcriptome.

| **Sample** | **Raw reads** | **Raw bases** | **Clean reads** | **Clean bases** | **Error rate (%)** | **Q30 (%)** | **GC content (%)** |
| --- | --- | --- | --- | --- | --- | --- | --- |
| QS9_CK1 | 45,181,148 | 6,822,353,348 | 44,728,740 | 6,459,694,515 | 0.024 | 98.4 | 95.2 |
| QS9_CK2 | 44,440,394 | 6,710,499,494 | 43,997,046 | 6,505,736,275 | 0.025 | 98.2 | 94.6 |
| QS9_CK3 | 44,555,858 | 6,727,934,558 | 44,032,356 | 6,528,401,745 | 0.025 | 97.9 | 93.9 |
| QS9_Cu1 | 44,833,248 | 6,769,820,448 | 44,192,696 | 6,395,208,219 | 0.025 | 98.2 | 94.7 |
| QS9_Cu2 | 45,510,570 | 6,872,096,070 | 44,994,860 | 6,509,401,876 | 0.024 | 98.3 | 94.9 |
| QS9_Cu3 | 43,894,986 | 6,628,142,886 | 43,192,096 | 6,238,775,781 | 0.025 | 98.0 | 94.3 |
| B1_CK1 | 46,378,814 | 7,003,200,914 | 45,830,642 | 6,553,812,774 | 0.025 | 98.3 | 94.8 |
| B1_CK2 | 47,129,308 | 7,116,525,508 | 46,545,016 | 6,705,006,570 | 0.024 | 98.4 | 95.2 |
| B1_CK3 | 44,825,362 | 6,768,629,662 | 44,293,356 | 6,426,864,371 | 0.024 | 98.5 | 95.3 |
| B1_Cu1 | 43,947,664 | 6,636,097,264 | 43,324,830 | 6,409,883,045 | 0.025 | 97.9 | 94.1 |
| B1_Cu2 | 44,924,764 | 6,783,639,364 | 44,224,070 | 6,439,673,327 | 0.026 | 97.8 | 93.9 |
| B1_Cu3 | 45,361,510 | 6,849,588,010 | 44,657,718 | 6,387,902,802 | 0.025 | 98.2 | 94.8 |
| B2_CK1 | 47,711,094 | 7,204,375,194 | 46,528,680 | 6,674,545,234 | 0.024 | 98.3 | 95.1 |
| B2_CK2 | 48,433,850 | 7,313,511,350 | 47,977,008 | 7,124,553,558 | 0.025 | 98.3 | 94.7 |
| B2_CK3 | 45,579,424 | 6,882,493,024 | 44,731,050 | 6,648,461,312 | 0.024 | 98.3 | 94.9 |
| B2_Cu1 | 45,822,308 | 6,919,168,508 | 45,260,392 | 6,722,895,304 | 0.024 | 98.3 | 94.9 |
| B2_Cu2 | 45,121,920 | 6,813,409,920 | 44,413,016 | 6,384,980,888 | 0.025 | 98.2 | 94.7 |
| B2_Cu3 | 44,231,874 | 6,679,012,974 | 43,773,478 | 6,414,096,304 | 0.025 | 98.2 | 94.6 |

CK stands for amoeba culturing on normal SM/5 agar plates, and Cu stands for amoeba culturing on SM/5 agar plates added with a high concentration of copper (50 mg/L). QS9 indicates uncolonized amoeba group, B1 indicates *P. agricolaris* colonized group and B2 indicates *P. hayleyella* colonized group. Q30 value represents the percentage of reads above quality score 30.

**Table S4.** Expressions of DEGs related to cell signaling and mitochondrial activities of uncolonized *D. discoideum*

| **Gene id** | **Gene name** | **Gene description** | **Regulation** | **Control group** | **Copper-treated group** |
| --- | --- | --- | --- | --- | --- |
| DDB_G0273397 | *carA-1* | G-protein-coupled receptor | up | 1.21 | 7.35 |
| DDB_G0273533 | *carA-2* | G-protein-coupled receptor | up | 1.38 | 8.00 |
| DDB_G0289073 | *csaA* | contact site A protein | up | 1.20 | 15.33 |
| DDB_G0279799 | *cprB* | cysteine protease | up | 0.81 | 17.55 |
| DDB_G0284331 | *regA* | cAMP phosphodiesterase | up | 5.55 | 13.44 |
| DDB_G0289145 | *pde7* | cAMP phosphodiesterase | up | 0.07 | 5.39 |
| DDB_G0279413 | *pkaR* | protein kinase A regulatory subunit | up | 14.76 | 37.54 |
| DDB_G0280689 | *tgrB1* | IPT/TIG domain-containing protein | up | 0.20 | 5.15 |
| DDB_G0280531 | *tgrC1* | signal protein | up | 0.05 | 2.84 |
| DDB_G0289329 | *dmtA* | des-methyl-DIF-1 methyltransferase | up | 1.40 | 8.35 |
| DDB_G0287101 | *noxB* | superoxide-generating NADPH oxidase flavocytochrome | up | 0.37 | 1.38 |
| DidioMr42 | *rnlA* | large subunit ribosomal RNA | down | 57.47 | 2.49 |
| DDB_G0292806 | *cluA* | 150 kDa protein | down | 15.95 | 6.61 |
| DidioMp11 | *atp1* | ATPase subunit 1 | down | 3.85 | 0.68 |

**Table S5.** Expressions of DEGs related to cell signaling and mitochondrial activity of *D. discoideum* colonized by different *Paraburkholderia* clades.

| **Gene id** | **Gene name** | **Gene description** | **Regulation** | **Control group** | **Copper-treated group** |
| --- | --- | --- | --- | --- | --- |
| ***P. agricolaris* colonized amoeba** | | | | | |
| DDB_G0273397 | *carA-1* | G-protein-coupled receptor | no significance | 0.67 | 1.99 |
| DDB_G0273533 | *carA-2* | G-protein-coupled receptor | no significance | 0.67 | 1.99 |
| DDB_G0284331 | *regA* | cAMP phosphodiesterase | no significance | 3.18 | 8.42 |
| DDB_G0279413 | *pkaR* | protein kinase A regulatory subunit | no significance | 7.14 | 20.07 |
| DDB_G0289329 | *dmtA* | des-methyl-DIF-1 methyltransferase | no significance | 0.83 | 1.81 |
| DidioMr42 | *rnlA* | large subunit ribosomal RNA | no significance | 7.36 | 5.61 |
| DidioMp11 | *atp1* | ATPase subunit 1 | no significance | 0.98 | 1.69 |
| ***P. hayleyella* infected amoeba** | | | | | |
| DDB_G0273397 | *carA-1* | G-protein-coupled receptor | no significance | 0.70 | 1.95 |
| DDB_G0273533 | *carA-2* | G-protein-coupled receptor | no significance | 0.74 | 2.09 |
| DDB_G0284331 | *regA* | cAMP phosphodiesterase | no significance | 3.43 | 5.03 |
| DDB_G0279413 | *pkaR* | protein kinase A regulatory subunit | no significance | 6.36 | 11.54 |
| DDB_G0289329 | *dmtA* | des-methyl-DIF-1 methyltransferase | no significance | 1.70 | 2.25 |
| DidioMr42 | *rnlA* | large subunit ribosomal RNA | no significance | 3.52 | 4.31 |
| DidioMp11 | *atp1* | ATPase subunit 1 | no significance | 0.68 | 1.07 |
